# Comparative Analysis of Changes of Myocardial Angiogenesis and Energy Metabolism in Postinfarction and Diabetic Damage of Rat Heart

**DOI:** 10.1155/2014/827896

**Published:** 2014-02-12

**Authors:** Sergey A. Afanasiev, Margarita V. Egorova, Dina S. Kondratyeva, Roman E. Batalov, Sergey V. Popov

**Affiliations:** FSBI “RI Cardiology” SB RAMS, 111a Kievskaya Street, Tomsk 634012, Russia

## Abstract

Comparative study of changes in myocardial activity of lactate dehydrogenase (LDH), succinate dehydrogenase (SDH), and capillary density distribution in the experimental models of diabetic and postinfarction damage of rat heart was performed. Data showed that decrease in LDH and SDH activities was observed in both pathologies which can suggest abnormal processes of glycolysis and oxidative phosphorylation in cardiac mitochondria. Activity of LDH and SDH in combined pathologies was comparative with the corresponding values of these parameters in control group. The authors hypothesize that these differences can be caused by specifics of myocardial vascularization. The results of the study showed that an increase in capillary density was found in all groups of rats with pathologies compared with control group. However, no significant differences in the intensity of angiogenesis processes were found between groups with pathologies.

## 1. Introduction

Myocardial hypertrophy is a typical response of the heart to changes in the conditions of cardiac functioning including the development of chronic pathologies. Disturbances in the energy metabolism at the cellular level represent the important stage of myocardial dysfunction development in hypertrophic cardiomyopathies [1]. The ATP formation and utilization processes are structured as the functional ensembles [2, 3]. Coordinated work of these ensembles directly depends on a state and structural changes of the cell itself. These ideas agree well with data suggesting that morphological changes in the cardiomyocytes seen in ischemic and diabetic cardiomyopathies accompany disturbances in the energy metabolism [4, 5]. These disturbances set up the pathologic circle leading to malignant development of the disease.

Normally, energy metabolism in the cardiomyocytes is supplied via the fatty acid and, partially, glucose oxidation. Development of the heart failure of ischemic genesis predisposes to oxidation of glucose which is more efficient in regard to oxygen consumption. In diabetes, fatty acids are the main substrate due to, first of all, the abnormal insulin-sensitive transport of glucose. In this, massive permeation of fatty acids into mitochondria leads to excessive accumulation of acetyl coenzyme A in them. This triggers inhibition of the pyruvate dehydrogenase complex [5]. However, the experimental and clinical data about the predominance of one or another type of energy production in chronic cardiac pathologies are controversial [5]. One of the adaptation myocardial reactions to the stimulated functioning in the adverse conditions is an upregulation of the apoptosis. Development of the microvessels can bridge the gap between oxygen demand and oxygen supply in the cardiac myocytes.

The aim of the experimental study was to perform a comparative analysis of chronic changes of the angiogenesis and energy metabolism in case of the postinfarction and diabetic heart damage.

## 2. Materials and Methods

The study was performed with adult male Wistar rats (250–300 g). The animals were assigned to four groups with 5 animals in each group: intact animals (control group or group 1); animals with myocardial infarction modeling (group 2); animals with induced diabetes (group 3); and animals with combined pathology such as myocardial infarction plus diabetes (group 4).

To model myocardial infarction, the etherized animals received ligature in the upper third of the left descending coronary artery [6]. The development of the postinfarction changes was verified morphologically. Follow-up examinations were carried out 6 weeks after coronary occlusion.

Diabetes mellitus was induced by a single intraperitoneal infusion of streptozotocin (Sigma, USA) in a dose of 60 mg/kg dissolved in 0.01 mol/L citrate buffer (pH 4.5) [6]. Manifestation of diabetes mellitus was verified if there were 3-fold to 4.5-fold increase in the concentration of blood glucose, body weight reduction, polyuria, and polydipsia. The animals were studied 6 weeks after the streptozotocin infusion. When the combined pathology was modeled, the animals received a single intraperitoneal infusion of streptozotocin in a dose of 60 mg/kg 4 weeks after coronary occlusion [6]. Follow-up studies were performed 2 weeks after the streptozotocin infusion (total 6 weeks of the pathology duration).

All animals were kept in the standard conditions of the animal resources facility. The animals were euthanized via the Rausch narcosis.

For the morphological examination, the fragments of the preserved left ventricular myocardium adjacent to scar tissue were dissected from the animals of group 2 and group 4. Anatomically similar samples were dissected from the hearts of the animals from group 1 and group 3. Fixation of the samples was done by using 10% neutral formalin. The histological processing in alcohols and paraffin embedding were performed according to the standard methods. To evaluate the status of myocardial vascularization, the histological sections (4 to 5 *μ*m) were stained with methenamine silver (P.A.S.M. reagent kit). For each section, the number of capillaries was counted in 10 random visual fields with 100-fold magnification and then the arithmetic mean was calculated.

Microscopic examinations of the sections at the light-optical level were performed by using the binocular microscope AxioLab A1 (Carl Zeiss, Germany).

For the histoenzymatic study, 10 *μ*m thick sections were used. The sections were prepared from rat frozen left ventricular myocardium by using the cryostat TP-OM-5-01 (Russia). Histochemical reactions were estimated to elucidate activity of succinate dehydrogenase, EC 1.3.99.1, (SDH) and L-lactate dehydrogenase, EC 1.1.1.27, (LDH) [7]. Quantitative assessment of enzymatic activity was done by using the luminescent microscope “Lyumam-IZ” (Russia) in transmitted light with the wave length of 546 nm in the areas of 0.5 *μ*m^2^. Optical density was measured in at least 50 individual cardiomyocytes.

Myocardial homogenate and mitochondria from cardiomyocytes of rats were obtained by the standard procedure of differential centrifugation in sucrose media containing: 250 mM sucrose, 10 mM EDTA, and 10 mM HEPES at pH 7.4 [8]. Mitochondria were suspended and stored in the solution containing 250 mM sucrose and 10 mM HEPES at pH 7.4.

Contents of free fatty acids (FFA) in blood plasma, myocardial homogenate, and mitochondrial suspension were determined enzymatically by the photoelectric colorimeter analyzer. Commercial kits of DiaSys Diagnostic Systems (Germany) were used to determine FFA contents.

Statistical processing of data was performed by using the applied software package STATISTICA 6.0. The studied parameters were not normally distributed (Shapiro-Wilk test, *P* > 0.05). Due to small group sizes and their independence of each other, statistical significance of values between groups was evaluated by using the nonparametric Mann-Whitney rank test. Within groups, statistical significance of differences was estimated via the Wilcoxon test for dependent variables. Single-factor analysis of variance was used for statistical processing of the morphological data. Values were considered statistically significant when *P* was < 0.05.

## 3. Results and Discussion

Data on FFA contents in the studied samples are presented in Table 1. You can see that an increase in FFA amount in blood plasma was found for all studied pathologies. However, no statistically significant differences were found between groups of animals with modeled pathologies. No significant differences in myocardial homogenate were observed between any groups. This result obviously suggests that cardiomyocytes preserve effective control over FFA uptake in conditions of the modeled pathologies. One can expect that this effect is present in real chronic diseases.

Essentially different result was obtained upon an estimation of FFA content in the mitochondrial suspension. All groups differed significantly from each other. The highest fatty acid content was found in group 3 (diabetes): the fatty acid level exceeded control values by more than 5 times. In group 2 (myocardial infarction), there was 3-fold increase in fatty acid content. However, in group of combined pathologies (group 4) this value was close to control (Table 1). It is quite possible that the found differences are characteristic of the differences in the mitochondrial capabilities to oxidize fatty acids in the studied pathologies.

Oxidation of FFA is an oxygen-dependent process. This fact enhances significance of the anaerobic energy production pathway in the pathological conditions. The results of the histological enzymatic study showed that cardiomyocyte lactate dehydrogenase (LDH) activity was significantly lower in rats with monopathologies (group 2 and group 3) than in control animals (Table 2). This perhaps is reflective of the decline in the anaerobic energy production process. However, LDH activity in the combination of pathologies was not statistically different relative to control group. This suggests that group 4 (combined pathology) preserves normal glycolysis processes.

Analysis of data showed a decrease in succinate dehydrogenase (SDH) activity by 1.5- and 2.6-times in group 2 and group 3, respectively, compared to group 1 (control) (Table 2). On the contrary, in group 4 (combined pathology), SDH activity was significantly higher by 2.5 to 3.5 times compared with monopathologies and control, respectively.

The results of our study suggested that the efficiency of both aerobic and anaerobic energy production pathways decreased in diseased myocardium when the pathological conditions developed as monopathology, separately from each other. However, when combined pathologies developed, the level of aerobic processes remained preserved whereas the aerobic pathway of ATP production was even elevated. Obtained data can be explained by the adaptive remodeling of energy metabolism. In particular, the process of energy production in cardiac mitochondria can switch to succinate dehydrogenase pathway of oxidation. According to the existing knowledge, the very succinate is subjected to active oxidation in tissues when there is lack of oxygen [9–11].

**Figure 1:**
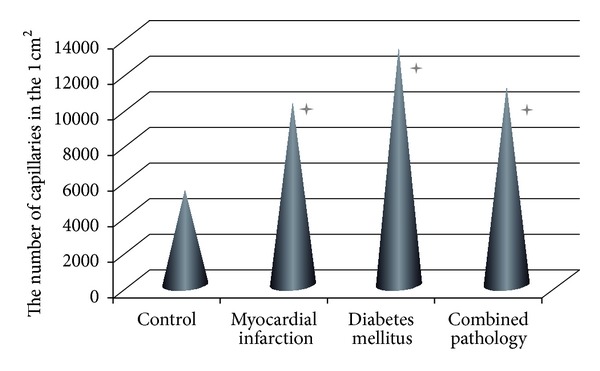
Distribution of capillary density in rat myocardium in case of mono- and combined pathologies. *P < 0.01 compared with control.

Augmentation of aerobic processes can be related to an activation of neoangiogenesis and, correspondingly, to increase in myocardial blood flow. In our study, the lowest density of capillaries was documented in control group (Figure 1). The highest capillary density was found in group 3. Its value statistically significantly exceeded the corresponding values in all other groups. In animals with combined pathologies, capillary density was significantly higher than in control animals and animals with myocardial infarction (Figure 1).

Therefore, the development of both pathologies and their combinations triggers the statistically significant increase in myocardial vascularization. However, angiogenesis occurs via the activation of different endogenous mechanisms in group 2 and group 3. The less pronounced myocardial vascularization in myocardial infarction in case of monopathology is probably caused by the local vascular bed damage and, correspondently, by formation of collaterals bypassing damaged part [7]. Multiorgan microangiopathies are characteristic of type 1 diabetes mellitus; this probably triggers the central mechanisms of angiogenesis and provides full-scale reaction. Indirect confirmation of this hypothesis is the fact that upregulation of neoangiogenesis is not seen in combined pathologies compared to diabetes. Moreover, capillary density is higher in case of combined pathology than in myocardial infarction alone (Figure 1).

Obtained data suggest that increase in myocardial capillary network plays an important role in upregulation of anaerobic energy production in combined pathology. However, we did not obtain evidence for a direct association between SDH activity and capillary density. Despite high density of capillaries, LDH activity was low in both groups of monopathologies, but high in case of combined pathology. There is no reason to claim that changes in intensity of aerobic and anaerobic processes of energy production in cardiac mitochondria are represented only by improvement of myocardial blood flow.

## 4. Conclusions 

The performed analysis suggests about normal activity of the aerobic and anaerobic processes of energy production in cardiomyocytes in case of the given combined pathologies. This aspect perhaps allows to simultaneously use glucose and fatty acids in a sufficiently enough way to provide the best heart functioning [4, 12]. Less pronounced abnormality of the energy metabolism is probably related to adaptive activation of SDH. This hypothesis does not contradict data suggesting that the changes in SDH activity as well as the changes in succinate formation and oxidation provide adaptation and preservation of the cellular energy production under stress [9, 10].

## Figures and Tables

**Table 1:** Fatty acid content in blood plasma, myocardial homogenate, and cardiac mitochondria from experimental rats (*X* ± *m*).

Groups of animals	FFA (nM/mg of protein)		
	Plasma	Homogenate	Mitochondria
1 “control”	0.38 ± 0.1	1.02 ± 0.14	0.83 ± 0.12
2 “myocardial infarction”	1.84 ± 0.04*	1.19 ± 0.14	2.86 ± 1.15^∗#^
3 “diabetes mellitus”	1.68 ± 0.11*	1.51 ± 0.17	5.83 ± 1.31^∗∧^
4 “myocardial infarction + diabetes mellitus”	1.54 ± 0.20*	1.35 ± 0.15	1.88 ± 0.78^∗#∧^

*P < 0.01 compared with control; ^#^ *P* < 0.01 compared with diabetes; ^∧^ *P* < 0.01 compared with myocardial infarction.

**Table 2:** Enzymatic activity in myocardium of animals from the observation groups (*X* ± *m*).

Animal groups	Enzymatic activity	
	LDH	SDH
1 “control”	0.73 ± 0.04	0.51 ± 0.02
2 “myocardial infarction”	0.41 ± 0.01*	0.32 ± 0.01^∗#^
3 “diabetes mellitus”	0.37 ± 0.03*	0.20 ± 0.02^∗∧^
4 “myocardial infarction + diabetes mellitus”	0.62 ± 0.03^#∧^	0.78 ± 0.05^∗#∧^

*P < 0.01 compared with control; ^#^ *P* < 0.01 compared with diabetes; ^∧^ *P* < 0.01 compared with myocardial infarction.
